# Spectroscopic Studies of Fluorescence Effects in Bioactive 4-(5-Heptyl-1,3,4-Thiadiazol-2-yl)Benzene-1,3-Diol and 4-(5-Methyl-1,3,4-Thiadiazol-2-yl)Benzene-1,3-Diol Molecules Induced by pH Changes in Aqueous Solutions

**DOI:** 10.1007/s10895-017-2053-y

**Published:** 2017-03-01

**Authors:** Arkadiusz Matwijczuk, Dariusz Kluczyk, Andrzej Górecki, Andrzej Niewiadomy, Mariusz Gagoś

**Affiliations:** 1 Department of Biophysics, University of Life Sciences in Lublin, Akademicka 13, 20-950 Lublin, Poland; 2 Department of Cell Biology, Institute of Biology, Maria Curie-Skłodowska University, 20-033 Lublin, Poland; 3 Department of Physical Biochemistry, Faculty of Biochemistry, Biophysics and Biotechnology of the Jagiellonian University, Gronostajowa 7, 30-387 Krakow, Poland; 4 Institute of Industrial Organic Chemistry, Annopol 6, 03-236 Warsaw, Poland; 5 Department of Chemistry, University of Life Sciences in Lublin, 20-950 Lublin, Poland

**Keywords:** Dual fluorescence effects, Molecular spectroscopy, Molecular aggregation, Substituent effects, 1,3,4-thiadiazole

## Abstract

This paper presents the results of stationary fluorescence spectroscopy and time-resolved spectroscopy analyses of two 1,3,4-thiadiazole analogues, i.e. 4-(5-methyl-1,3,4-thiadiazol-2-yl)benzene-1,3-diol (C1) and 4-(5-heptyl-1,3,4-thiadiazol-2-yl)benzene-1,3-diol (C7) in an aqueous medium containing different concentrations of hydrogen ions. An interesting dual florescence effect was observed when both compounds were dissolved in aqueous solutions at pH below 7 for C1 and 7.5 for C7. In turn, for C1 and C7 dissolved in water at pH higher than the physiological value (mentioned above), single fluorescence was only noted. Based on previous results of investigations of the selected 1,3,4-thiadiazole compounds, it was noted that the presented effects were associated with both conformational changes in the analysed molecules and charge transfer (CT) effects, which were influenced by the aggregation factor. However, in the case of C1 and C7, the dual fluorescence effects were visible in a higher energetic region (different than that observed in the 1,3,4-thiadiazoles studied previously). Measurements of the fluorescence lifetimes in a medium characterised by different concentrations of hydrogen ions revealed clear lengthening of the excited-state lifetime in a pH range at which dual fluorescence effects can be observed. An important finding of the investigations presented in this article is the fact that the spectroscopic effects observed not only are interesting from the cognitive point of view but also can help in development of an appropriate theoretical model of molecular interactions responsible for the dual fluorescence effects in the analysed 1,3,4-thiadiazoles. Furthermore, the study will clarify a broad range of biological and pharmaceutical applications of these compounds, which are more frequently used in clinical therapies.

**Electronic supplementary material:**

The online version of this article (doi:10.1007/s10895-017-2053-y) contains supplementary material, which is available to authorized users.

**Graphical Abstract:**
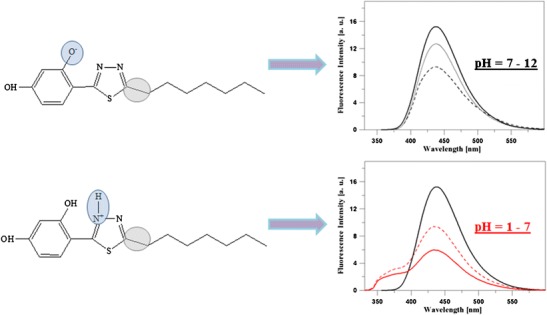
Upper left corner – C7 molecule at high pH, right upper corner - fluorescence emission spectrum for C7 dissolved in H_2_O at high pH (7–12) - single fluorescence. Bottom left corner – C7 molecule at low pH (1–7), lower right corner - fluorescence emission spectrum for C7 dissolved in water at low pH – two fluorescence emissions. The circles indicate the group related to dissociation of molecules at low and high pH and the additional long circles indicate C1 or a molecule with a shorter acyl chain.

## Introduction

As reported by the World Health Organisation, one of the major challenges of modern medicine is the fight against cancer and neurodegenerative diseases. According to literature data, these diseases are currently the leading causes of patients’ mortality worldwide [1, 2]. Great hopes in the fight against cancer and neurodegenerative diseases are placed on synthetic compounds from the 1,3,4-thiadiazole group, whose different derivatives are already known and clinically applied. The 1,3,4-thiadiazoles with a substituted resorcyl fragment analysed in this study are highly promising compounds in anticancer and neurodegenerative diseases. As indicated in most research papers, 1,3,4-thiadiazoles are the most attractive neuroprotective-activity molecule system of all the other thiadiazole systems [3–5]. Many compounds of this group have been used in medicine as e.g. oxidation inhibitors, colourants, and metal complexing compounds [6]. Additionally, the literature shows that the thiadiazole family comprises compounds with antitumour [7–10], antifungal [11], antibacterial [11], anti-inflammatory [12], anticonvulsant [13], antiviral [14], antituberculosis [15], antihypertensive [16], and antidepressant [17] activity.

**Scheme 1:**
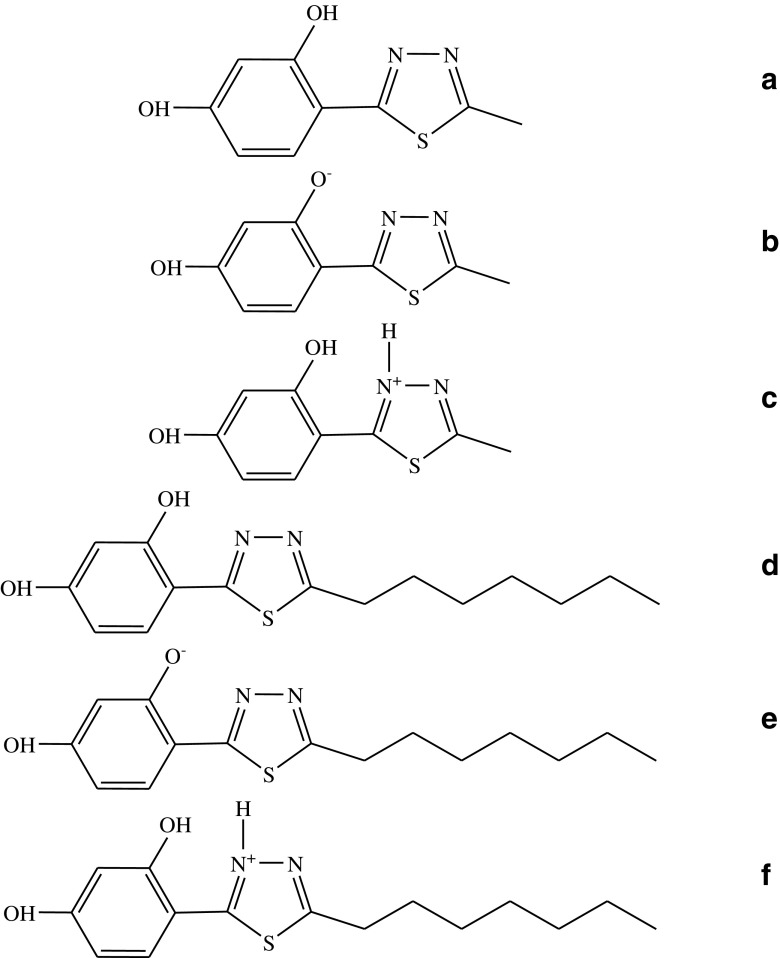
Chemical structure of the C1 molecule (**a** – enol form, **b** – form ionised with the –O^−^ group, **c** – form ionised with the–N^+^–H group) and C7 molecule (**d** – enol form, **e** – form ionised with the –O^−^ group, **f** – form ionised with the–N^+^–H group)

Two highly promising 1,3,4-thiadiazole analogues with a confirmed neuroprotective activity, i.e. 4-(5-methyl-1,3,4-thiadiazol-2-yl)benzene-1,3-diol (C1) (C1, Scheme 1A–C) and 4-(5-heptyl-1,3,4-thiadiazol-2-yl)benzene-1,3-diol (C7) (C7, Scheme 1D–F), were selected in this study for the investigations of the mechanism of molecular interactions. Noteworthy, these 1,3,4-thiadiazole compounds exhibit not only remarkable and confirmed pharmacological properties but also very interesting spectroscopic traits, which contribute to their biological activity. The spectroscopic effects exhibited by the 1,3,4-thiadiazole analogues include e.g. the effects of keto/enol tautomerism induced by changes in medium polarizability [18–21], effects associated with crystal polymorphism [22] and solvatomorphism [23], and interesting interactions in model lipid systems [24, 25]. Additionally, the analysed compound group exhibits an interesting dual fluorescence effect or an effect of several fluorescence spectra, depending on the concentration [26], pH, or changes in the medium temperature, which will be presented in this paper [27]. Moreover, these compounds are good ligands, which can form complexes with d-block metal ions [28], and can therefore be used in novel medical applications. The combination of the spectroscopic and crystallographic effects reported in the papers cited above has great importance for elucidation of the broad spectrum of the pharmacological activity of these compounds.

The aim of the present spectroscopic study was to investigate C1 and C7 in a medium with different concentrations of hydrogen ions and to describe the dual fluorescence effect observed in these molecules. In previous studies, the dual fluorescence effect was observed for one representative of the analogue group (FABT) [27]. However, in this study, the effect was observed in a different (substantially higher) energy range, which completely changed the photophysical properties of the analysed molecules. Using spectroscopic approaches, such as the electron absorption spectroscopic technique, fluorescence methods combined with the RLS technique, and mainly measurements of fluorescence lifetimes, we have shown the complexity of physical processes that exert an impact on the observed effects. The dual fluorescence effects can be induced in different molecules by changes in medium polarity, pH of the solution, temperature, or the concentration of the sample [29–33]. According to theories attempting at elucidation of the observed fluorescence effects, they can be related to appearance of intramolecular CT states [34] and the so-called TICT states (Twisted Intramolecular Charge Transfer) [35, 36]. Another explanation of the analysed phenomena is the process of Excited-State Intramolecular Proton Transfer ESIPT [37–42]. Other theories postulate formation of compound concentration-induced excimer systems [43, 44], various types of acid-base reactions in the investigated system, or very frequent formation of excited-state tautomeric systems [45, 46]. The anti-Kasha mechanism of the dual fluorescence effect postulated in 2015 in the Journal of Physical Chemistry B by Brancato et al. should also be mentioned [47]. However, as suggested by the research results presented in this paper, none of these mechanisms seems to be successful in elucidation of the observed effects and molecular mechanisms involved in the spectroscopic changes.

The studies performed with the use of stationary fluorescence spectroscopy and time-resolved spectroscopy in an aqueous medium with different pH values revealed the dual fluorescence effect in both analogues.

Formulation of a concise theory for the dual fluorescence effects observed in the 1,3,4-thiadiazole analogues is crucial for understanding their biological activity. The effect is attractive for theoretic reasons, as it can be used for examination of the excitation state in various molecular transformations and for designing new molecular probes of (dual) fluorescence.

## Material and Methods

### Materials

4-(5-methyl-1,3,4-thiadiazol-2-yl)benzene-1,3-diol (C1) (see Scheme 1D) and 4-(5-heptyl-1,3,4-thiadiazol-2-yl)benzene-1,3-diol (C7) were synthesized in the Department of Chemistry of the University of Life Sciences in Lublin, details of the procedure are described elsewhere [3]. The purification procedure of the C1 and C7 compound is described in detail in the references [3]. The concentration of the compounds was c = 1.25 × 10^−6^ M for C1 and c = 1.19 × 10^−6^ M for C7.

### Methods

#### pH Measurement and Preparation

All solutions were measured with an Elmetron CP-502 pH-meter at room temperature. In the case of the aqueous C1 and C7 solutions, 0.1 M NaOH was first added to water to obtain pH 12. Afterwards, powder C1 and C7 was dissolved therein. Next, 0.1 M HCl acid was slowly added to obtain a certain pH value in the water C1 and C7 solution. The pH was continually controlled. Respective titration curves for both 1,3,4-thiadiazole analogues are shown in Fig. S1 in the Supplementary Materials. The insets in Fig. S1 present ionised functional groups and pK points for both compounds (see below).

#### Electronic Absorption Spectroscopy

Electronic absorption spectra of C1 and C7 were recorded on a double-beam UV-Vis spectrophotometer Cary 300 Bio (Varian) equipped with a thermostatted cuvette holder with a 6 × 6 multicell Peltier block. Temperature was controlled with a thermocouple probe (Cary Series II from Varian) placed directly in the sample. All experiments were carried out at 23 °C. The spectral slit width was 1.5 nm in the measurements of the electron absorption spectra.

#### Electronic Fluorescence Spectroscopy with the RLS Technique

Fluorescence excitation and emission as well as synchronous spectra were recorded with a Cary Eclipse spectrofluorometer (Varian) at 23 °C. Fluorescence spectra were recorded with 0.5 nm resolution and corrected for the lamp and photomultiplier spectral characteristics. Resonance light scattering (RLS) measurements were performed as in Pasternack and Collings [48, 49]. The excitation and emission monochromators of the spectrofluorimeter were scanned synchronously (0.0 nm interval between excitation and emission wavelengths), the slits were set to obtain spectral resolution of 1.5 nm. The spectral analysis was performed with the use of Grams/AI 8.0 software (Thermo Electron Corporation).

#### Time-Correlated Single Photon Counting (TCSPC)

Time-correlated single photon counting (TCSPC) measurements were performed on a FluoroCube fluorimeter (Horiba, France). The samples were excited with a pulsed NanoLED diode at 372 nm (pulse duration of 150 ps) operated with 1 MHz repetition. To avoid pulse pile-up, the power of the pulses was adjusted to an appropriate level using a neutral gradient filter. Fluorescence emission was recorded using a picosecond detector TBX-04 (IBH, JobinYvon, UK). The DataStation and the DAS6 software (JobinYvon (IBH, UK)) were used for data acquisition and signal analysis. All fluorescence decays were measured in a 10 × 10 mm quartz cuvette, using an emitter cut-off filter with transmittance for wavelengths longer than 408 nm. The excitation profiles required for the deconvolution analysis were measured without the emitter filters on a light scattering cuvette. All measurements were performed in water at 20 °C and various pH. Fluorescence decay was analysed with a multiexponential model given by the equation:

1 $$ {I}_t=\sum_i{\alpha}_i \exp \left(-\frac{t}{\tau}\right) $$

where α_i_ and τ_i_ are the pre-exponential factors and the decay time of component *i*, respectively.

Best-fit parameters were obtained by minimization of the reduced χ [2] value as well as residual distribution of experimental data. The average lifetime of fluorescence decay was calculated according to the following equation:

2 $$ \left\langle \tau \right\rangle =\frac{\underset{i}{\Sigma}{\alpha}_i{\tau}_i^2}{\underset{i}{\Sigma}{\alpha}_i{\tau}_i} $$

## Results and Discussion

By plotting the pH titration curves for C1 and C7, characteristic pK points for the ionisation-associated groups were determined in the studied 1,3,4-thiadiazoles (Fig. S1 in the Supplementary Materials). For the –O^−^ group in the ortho position in the resorcyl ring, pK_C7_ = 8.8 and pK_C1_ = 8.1 (Fig. S1 in the Supplementary Materials - figure insets) and for the –NH^+^ group, pK_C7_ = 4.9 and pK_C1_ = 4.1 (Fig. S1 in the Supplementary Materials - figure insets).

**Fig. 1:**
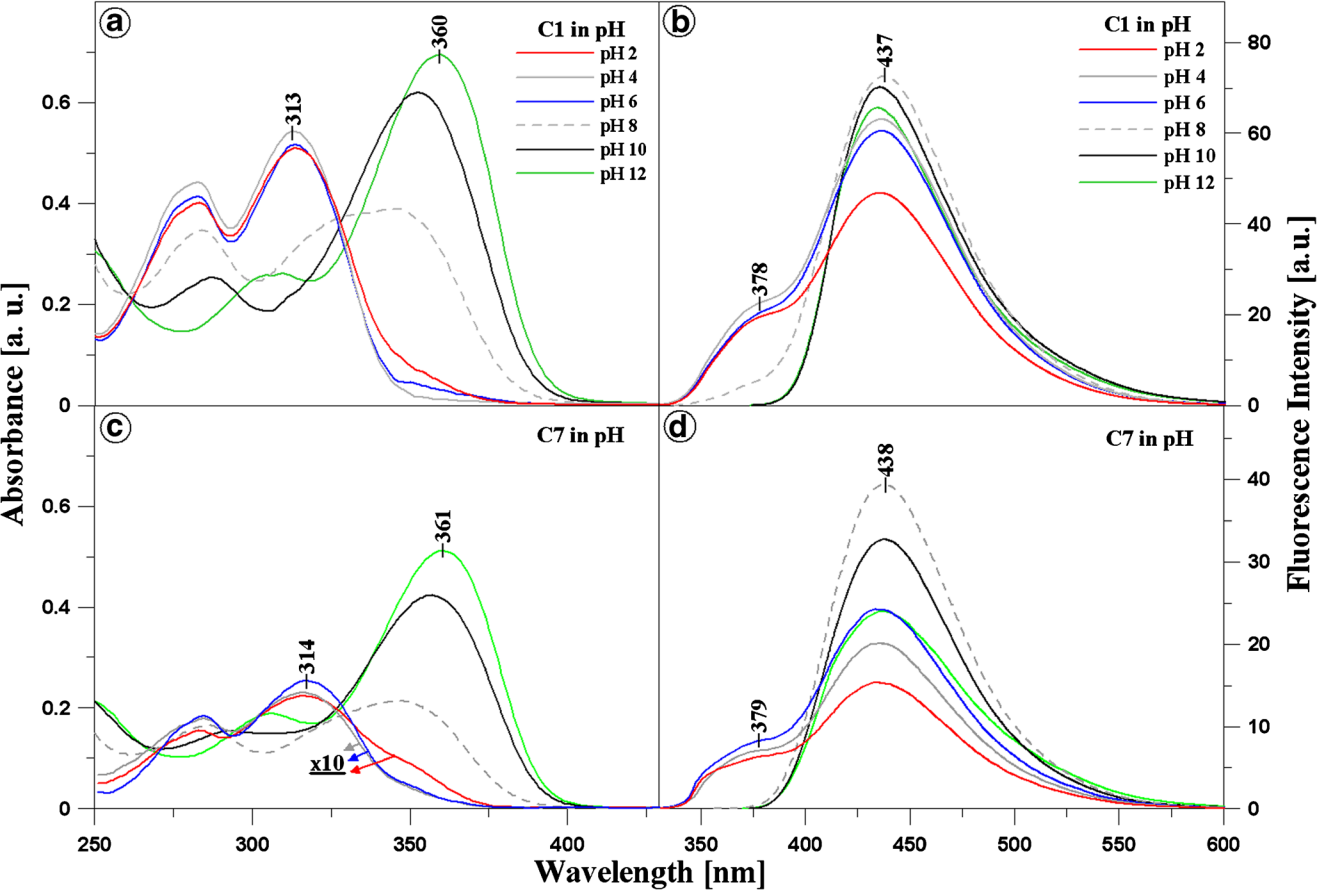
Panels A and C present electron absorption spectra for C1 (*Panel A*) and C7 (*Panel C*) generated in the aqueous solution at pH 12, 10, 8, 6, 4, and 2. For C7 in Panel C, the absorption spectra at pH 2, 4, and 6 were multiplied by 10 for better presentation and comparison of the analysed effects. Panels B and D show fluorescence emission spectra corresponding to the spectra from Panels A and C for C1 (*Panel B*) and C7 (*Panel D*). The excitation wavelength corresponded to the maximum of the respective absorption band

**Fig. 2:**
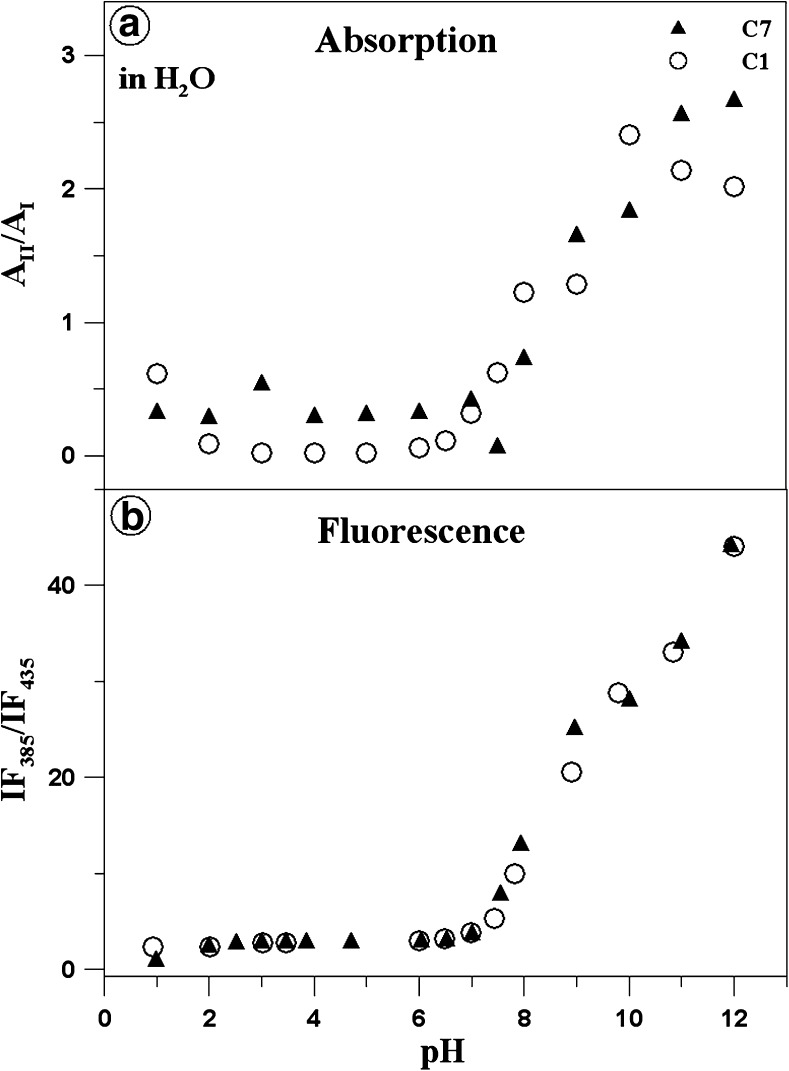
Panel A presents the ratio of the maximum electron absorption at ca. 360/313 nm for C1 (*white circles*) and at 361/314 nm for C7 (*black triangles*), i.e. the ratio between the predominant monomeric form (ionised with the –O^−^ group) and the predominant associated form for a given compound (ionised with the –N-H^+^ group) depending on the pH of the aqueous solution. Panel B shows the ratio of the fluorescence emission intensity for C1 (*white circles*) and C7 (*black triangles*) depending on changes in the pH of the aqueous solution. The points were read from the absorption and fluorescence emission spectra presented in Fig. 1. The measurements of the absorption and electron fluorescence spectra, from which the respective absorption and fluorescence maxima were read

Panels A and C in Fig. 1 present results obtained with electron absorption spectroscopy for C1 (Panel A) and C7 (Panel C) over the entire range of hydrogen ion concentrations (from pH 1 to pH 12). There results show distinct changes in the shape of the spectra, in particular in the region that is relevant to the physiological values. Spectra for pH 2, 4, 6, 8, 10, and 12 for both analysed compounds are presented for better clarity. The spectra in the pH range from 1 to 6 were multiplied by 10 (specifically: for C7 at pH 2, 4, and 6) for clearer presentation of the analysed effects for C7 (Panel C in Fig. 1). As shown in both panels in Fig. 1, the dissociation of the –OH group from the resorcyl ring in the *ortho* position (Scheme 1B and E) results in a clear hypsochromic shift by 301 cm^−1^ in the case of C7 and by 303 cm^−1^ for C1 in the compound spectra at pH 12. In addition, there is a bathochromic shift by 3845 cm^−1^ for C7 and 3864 cm^−1^ for C1 for spectra of both compounds at pH 1, compared with their spectra at pH 7. These shifts are used for calculation of the distances between the molecules in the dimer using the exciton splitting theory. In both 1,3,4-thiadiazole analogues, the ionisation process is accompanied by compound aggregation [27]. At pH ca. 7–8, distinct broadening of the absorption spectra is evident for C1 and C7 (dashed grey line in Panels A and C, Fig. 1), which indicates a probability of the presence of other than monomeric spectral forms of the analysed structures [27]. In the case of the C7 spectrum at pH 2, the absorbance is the lowest, which implies substantial predominance of aggregated forms in this compound (see Panel B in Fig. 2). In turn, in the case of C1, the lowest absorbance intensity is observed in the same range, but it is substantially higher than that for C7 at the corresponding pH. This indicates an impact of the structure of the analysed compounds, in particular the structure of their alkyl substituents, on the mode of formation of aggregated forms of these molecules. The structure of substituent groups can have a significant influence (through processes related to stronger aggregation) on the solubility of the analysed molecules in different organic solvents. For C1 at pH 4, the compound absorbance unexpectedly increases slightly, suggesting an increase in the number of the monomeric forms of this analogue. No such phenomenon is observed in the case of C7 in the specified concentration range, which evidences stronger interactions between C7 molecules and formation of more durable aggregates. In C7, a remarkable decrease in the absorbance level is visible, which implies very strong aggregation at a (probably) constant level along the decrease in the pH value (at low pH).

Based on the exciton splitting theory and the spectral shifts presented in Fig. 1 and above (and in Fig. S2 in the Supplementary Materials), it was possible to calculate the distance between adjacent chromophores of molecules C1 and C7 in the dimeric structure [49]. Fig. S2 (in the Supplementary Materials) shows electron absorption spectra for C7 (Panel A) and C1 (Panel B) normalised at the maximum. Underpinning of the band can be observed for both C1 and C7 at pH ca. 1, compared with pH 7. For C7 (Panel A), the band with a maximum at ca. 319 nm (31,348 cm^−1^) at pH 7 is slightly shifted towards 317 nm (31,546 cm^−1^) and a band with a maximum at ca. 351 nm (28,490 cm^−1^) appears on the longwave side. Similarly, in the case of C1, the main bands with a maximum at ca. 316 nm (31,646 cm^−1^) is shifted at low pH values towards ca. 314 nm (31,847 cm^−1^) and a band with a maximum at ca. 357 nm (28,011 cm^−1^) can be seen on the longwave side.

Based in the exciton splitting theory, the distance between adjacent chromophores R_β_ can be calculated using the formula:

3 $$ {R}_{\beta}=1.71\sqrt[3]{\frac{\mu^2\kappa}{\eta^2\beta}} $$

where μ is the diploe moment of transition of the interacting molecules, η – refractive index, β – dipole-dipole interaction energy (in a classical approach). In the excitonic model, one can consider an aggregated structure formed through interaction of identical molecules, in which transition dipole moments of adjacent molecules are parallel, hence α = 0 (where, κ = 1 - 3cos [2]θ, where θ is the angle between the transition dipole moments). As proposed in the exciton splitting theory [50, 51], κ = 1 for the *card pack* molecule arrangement in the aggregate and κ = −2 for the *head to tail* aggregate. The transition dipole moment calculated via integration of the absorption spectrum is μ = 4.16 D (in H_2_O) and μ = 4.25 D for C7 (the values of transition dipole moments in other solvents and water as well as the molar value of the extinction coefficients are presented in Table S1 in the Supplementary Materials). The calculated distance between adjacent chromophores is 4.29 Å ^50^ in the case of the C1 dimers in an aqueous solution and 3.87 Å for C7. These results are consistent with crystallographic data presented in some other 1,3,4-thiadiazole compounds [27]. The distance in crystals is lower than in solutions, which is the cause of the obviously denser packing of the analysed molecules in the crystalline structure and stronger intermolecular interactions. However, the most important fact is that the distance between adjacent molecules in C7 is substantially smaller than in C1.

Panel A in Fig. 2 shows the ratio of the absorbance maximum at 360 nm (predominant monomeric form, Scheme 1B, and E – ionised with the –O^−^ group) and 313 nm (predominant associated form, Scheme 1C, and F – ionised with the–NH^+^ group) for C1 (open circles) as well as the ratio of the absorbance maximum of 361 nm and 314 nm for C7 (black triangles), depending on the pH of the aqueous solution. The predominance of the negatively ionised form (predominance of the monomeric form) for both C1 and C7 compounds is most evident at the high pH values (pH 12 for C7 and pH 10.5 for C1). In contrast, the predominance of the forms ionised with the NH^+^ group can be observed at the low pH values (at pH 1 for both C7 and C1). In an acidic environment, the presence of a positively charged NH^+^ group in the thiadiazole ring should also lead to monomerisation. However, the presence of the –OH groups in the resorcyl ring facilitates generation of hydrogen bonds not only with water but also with other molecules, resulting in their aggregation. In turn, the greatest changes in the presented ratio in both compounds can be observed for the physiological pH values, which can be clearly seen in the absorption spectra presented in Panels A and C of Fig. 1.

In the next step of the investigations, the compounds were analysed with the fluorescence spectroscopy methods. Noteworthy are the effects presented in Panels B and D of Fig. 1. The panels show fluorescence emission spectra for C1 (Panel B) and C7 (Panel D), corresponding to the absorption spectra presented in Fig. 1 (Panels A and C), together with the change in the pH value of the aqueous solution (pH 2, 4, 6, 8, and 10 are presented analogously as for the absorption spectra). The excitation wavelength for all the analysed samples corresponds to the maximum of respective absorption bands. A dual fluorescence effect with a maximum at ca. 380 nm and 440 nm in the pH range from 1 to 7.5 (Panels C and D) is evident in the case of both compounds. Above pH 7.5, single fluorescence with a maximum at 438 nm for C7 and 437 nm for C1 is observed. At pH 8, only slight underpinning of the respective fluorescence emission spectrum is noted for C1. Additionally, it should be emphasised that the intensity of the fluorescence emission spectra for C7 is substantially lower than that for C1 in the range where the aforementioned effect can be observed (the same concentration of both analogues). This clearly indicates a considerably greater degree of aggregation in C7 than that in C1 and more intensive fluorescence emission decay. This was also observed in the calculations based on the exciton splitting theory (see above), where the distances between adjacent chromophores were markedly smaller in C7 than in C1.

Panel B in Fig. 2 shows the ratio of short- and longwave fluorescence emission intensity for both compounds (emission with a maximum of ca. 380 nm vs. emission with a maximum of ca. 440 nm) depending on the pH changes in the aqueous solution. Evidently, in the pH range from 1 to 7.5 for C7 and C1, the points are arranged in one almost horizontal line, likewise for the spectra shown in Fig. 1 (Panels A and C). This implies that the process of nitrogen atom protonation is in equilibrium and does not change the intensity of the ratio in the analysed 1,3,4-thiadiazole analogues (Scheme 1C and F). At a pH value higher than 7.5 (for both compounds), the ratio increases significantly and the fluorescence at 378/9 nm disappears for both compounds.

Noteworthy, a longwave fluorescence band with a maximum at ca. 500 nm appeared in the case of another 1,3,4-thiadiazole described previously, i.e. FABT [27], in which the structural differences are related to the presence of the N-H group and a fluorobenzene ring. In turn, in the compounds analysed in this paper, the decrease in the pH value is accompanied by a shortwave fluorescence emission band with a maximum at ca. 380 nm. Therefore, changes in the energy structure of the 1,3,4-thiadiazole molecules are observed. This effect can be associated with the presence of the amino N-H group in the FABT structure located at the 1,3,4-thiadiazole ring [27]. In the case of a 1,3,4-thiadiazole that structurally resembles FABT, although without the fluorobenzene fragment, and differs from C1 only in the N-H group, a band with a maximum at ca. 380 nm is observed at high pH values (results submitted for publishing). In turn, acidification yielded a separate band at ca. 440 nm (as in the case of C1 and C7 at the high pH values). These considerations indicate an impact of the substituents in the 1,3,4-thiadiazole structure on the position of fluorescence bands despite the identical chromophore organisation (five conjugated double bonds) in the molecule.

### Fluorescence Lifetime Study

**Fig. 3:**
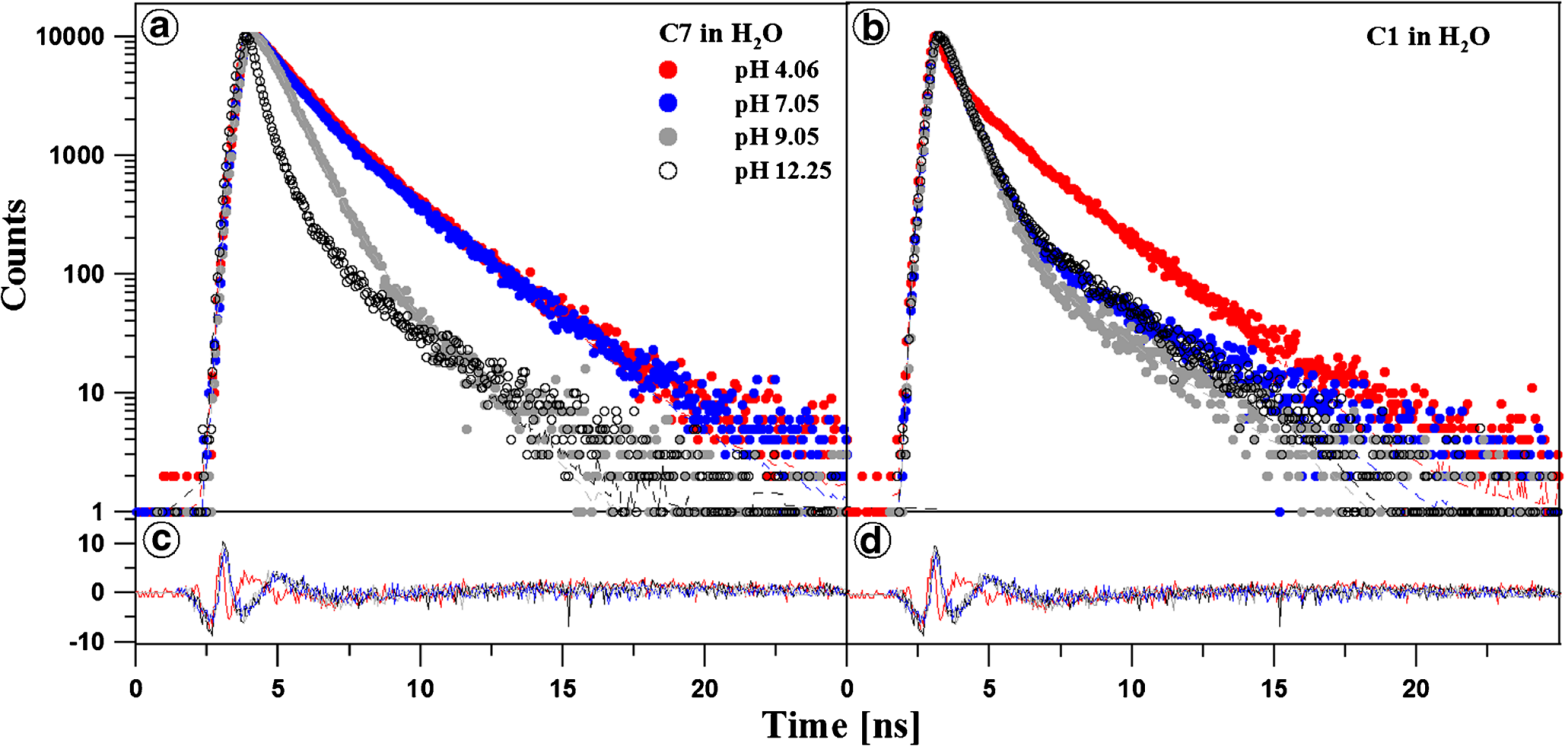
The pH effect of the fluorescence decay of C7 and C1 in water. Dotted curves show the decay of fluorescence emission observed using the TCSPC technique at a given pH for C7 and C1 in Panels A and B, respectively. Solid lines are best exponential fits. The excitation pulse profile, set up at 372, is shown by the dotted black curve. Residuals determined for the presented fits are shown below the decay curves in Panels C and D

**Fig. 4:**
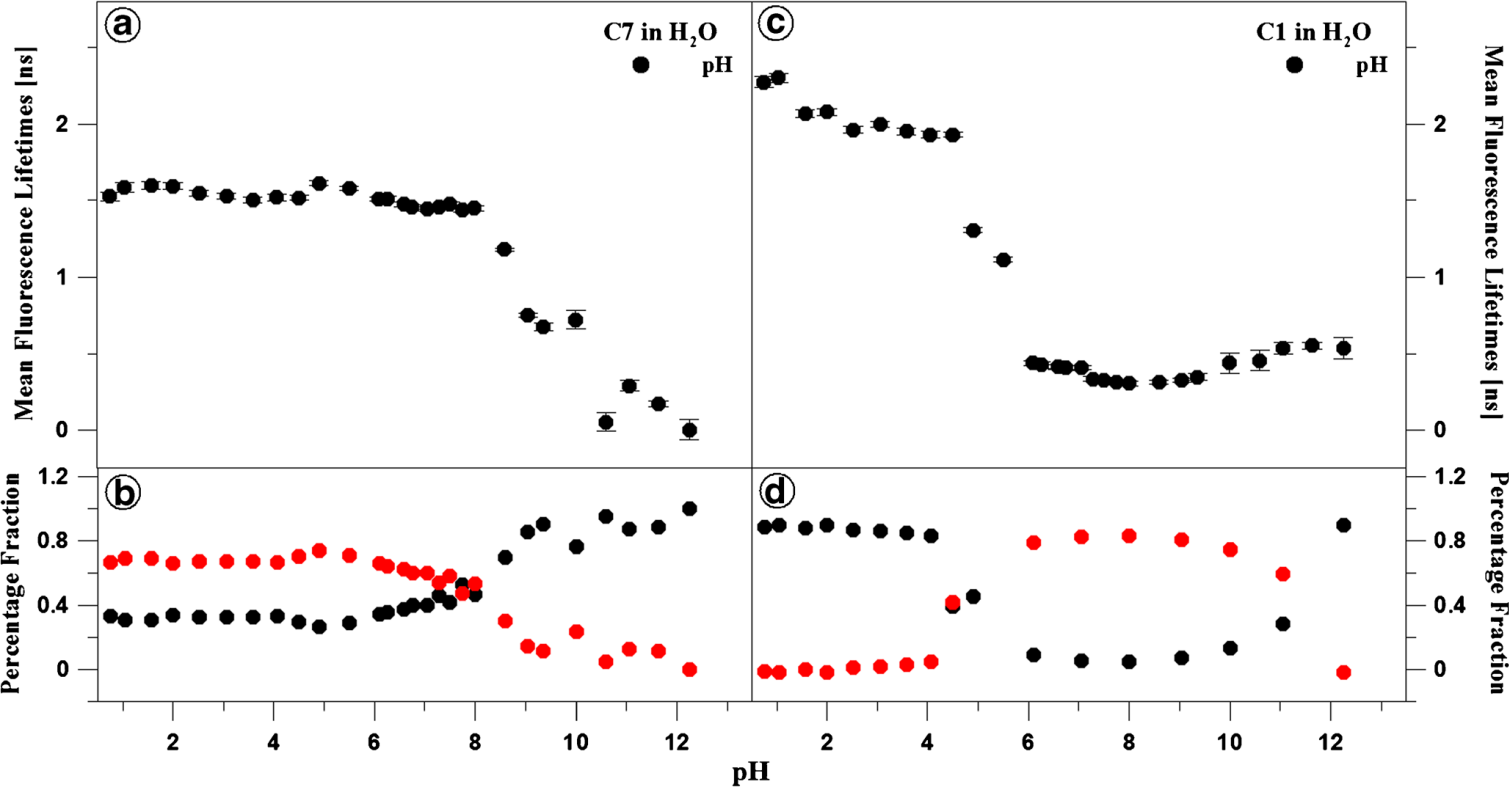
The effect of pH on the fluorescence lifetime for C7 (*Panels A and B*) and C1 (*Panels C and D*). The dependence of the mean fluorescence lifetimes on the best exponential fits at given pH are shown in Panels A and C, while intensities of the fractional fluorescence lifetimes are presented in Panels B and D

Figures 3 and 4 as well as Tables 1 and 2 present the results of measurements of fluorescence lifetimes for C1 and C7 in the aqueous solution over the entire pH range (shown for pH 4, 7, 9, and 12). Light with a wavelength of 372 nm was used for excitation. At pH 7, the selected excitation wavelength is typical for the longwave absorption edge of the monomeric form and resonant excitation of the aggregated form (~370 nm). Fluorescence decay was monitored with the TCSPC method at a temperature of 22 °C for light with a wavelength over 408 nm. The results were analysed by deconvolution of the fluorescence decay using Eq. (1) each time for *i* = 1, 2, and 3. In a majority of the analysed cases, the double exponential model of fluorescence decay proved to be optimal. The mono-exponential decay model was insufficient, and addition of a third component did not improve the quality of the fit, which was verified by the value of the fit parameter and analysis of residue distribution (Fig. 3, Panels C and D). A single exponential was only observed for C1 at pH lower than 5. In these cases, an additional component did not improve the fit. For C7, both components of the fluorescence lifetimes exhibited very little variability at pH below 8,5. In this range, the lifetime of the longer component is between 2 and 2,5 ns and is rapidly reduced to ca. 1 ns over the threshold pH value. Its contribution is ca. 70% at the low pH values and declines to ca. 10% at the higher pH values. The lifetime of the second component is ca. 0.5 ns at the lower pH values and decreases to several tens of ps for the higher pH values. As a result of this variability, the average lifetime is ca. 1,6 ns for pH lower than 8 and is reduced to several tens of ps at the higher pH values. In the case of C1, the component with the longer lifetime has a similar value to that observed for C7 and is 2–2,5 ns over the entire pH range. From pH 5, a second component with a lifetime of 0,5 ns is observed and its value changes insignificantly over the analysed pH range. With the additional component, the average lifetime is markedly reduced at the pH increase up to ca. 6 and then remains relatively constant at ca. 0,5–1 ns up to the pH value of 11.

**Table 1:** Fluorescence lifetimes of C7 in H_2_O in relation to changes in pH

**C7**			
pH	τ	±	Δτ
0.8	1.59	±	0.03
1.0	1.59	±	0.03
1.6	1.60	±	0.03
2.0	1.59	±	0.02
2.5	1.55	±	0.02
3.1	1.53	±	0.02
3.6	1.51	±	0.02
4.1	1.52	±	0.02
4.5	1.52	±	0.02
4.9	1.61	±	0.02
5.5	1.58	±	0.01
6.1	1.51	±	0.01
6.3	1.51	±	0.02
6.6	1.47	±	0.01
6.8	1.46	±	0.01
7.1	1.44	±	0.01
7.3	1.46	±	0.01
7.5	1.48	±	0.01
7.8	1.44	±	0.01
8.0	1.45	±	0.01
8.6	1.18	±	0.01
9.1	0.75	±	0.01
9.4	0.68	±	0.02
10.0	0.72	±	0.06
10.6	0.12	±	0.10
11.1	0.29	±	0.03
11.6	0.17	±	0.02
12.3	0.11	±	0.07

**Table 2:** Fluorescence lifetimes of C1 in H_2_O in relation to changes in pH

**C1**			
pH	τ	±	Δτ
1.0	2.45	±	0.06
2.0	2.24	±	0.03
3.0	2.17	±	0.01
4.0	2.15	±	0.01
5.0	1.29	±	0.01
6.0	0.73	±	0.01
7.0	0.63	±	0.01
8.0	0.60	±	0.01
9.0	0.62	±	0.01
10.0	0.72	±	0.01
11.0	0.81	±	0.04
12.0	0.81	±	0.04

Noteworthy, drastic changes in the length of the lifetimes are observed from a pH value of ca. 8 in the case of C7 (Fig. 4, Panel A) and from pH of ca. 5.5 in C1 (Fig. 4, Panel C). Similarly, the percentage proportion of the components of the average lifetimes for C1 and C7 changes at exactly the same pH values (see Fig. 4, Panels B and D). This clearly indicates substantially stronger aggregation of the C7 molecules, compared with C1, which has already been observed in the absorption spectra (Fig. 1, Panels A and C), where a significant reduction in the absorbance level was noted. This proves considerably better solubility of C1 in the aqueous medium with pH relevant from the physiological point of view.

The lengthening of the fluorescence lifetime in the case of molecules exhibiting the dual fluorescence effect is characteristic for charge-transfer systems and excimers [52], in contrast to processes induced by the phenomenon of aggregation (dimerisation), in which a clear reduction in the lifetime [27] or decay is observed.

### Resonance Light Scattering Study

**Fig. 5:**
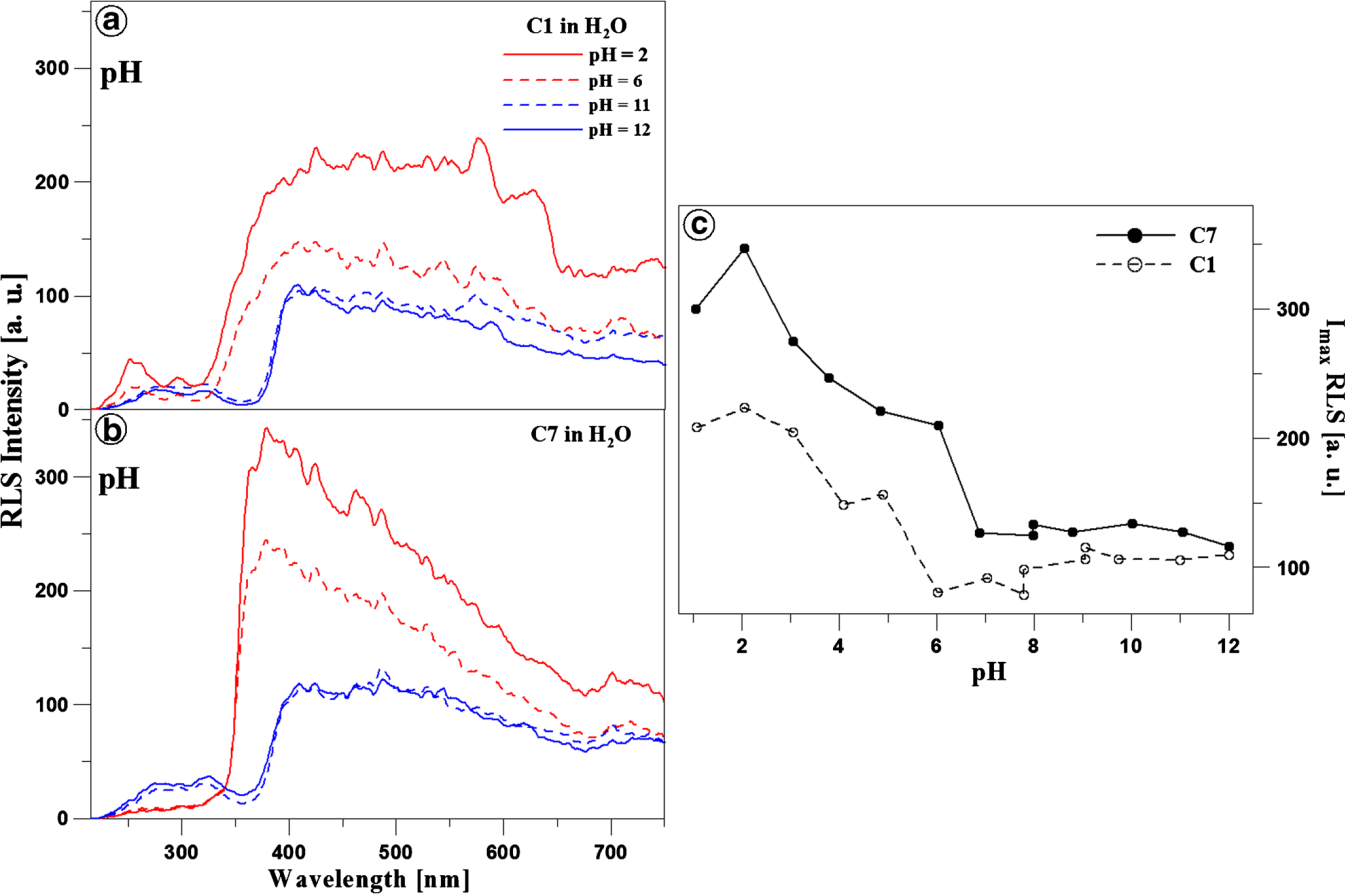
Panels A and B: RLS spectra (Resonance Light Scattering, Δλ = 0) for C1 and C7 obtained in the aqueous solution at the different pH values, presented in the figure for pH 1, 3, 7, and 10, respectively. Panel C shows the ratio of the intensity of the RLS spectra for C1 (*black line*) and C7 (*dashed grey line*) depending on the pH of the aqueous solution. All RLS spectra were obtained at *T* = 23 °C

In order to relate the fluorescence effects observed with the molecular aggregation effects, respective Resonance Light Scattering (RLS, Δλ = 0) spectra were obtained for C1 and C7 in the aqueous solution, depending on the changes in the medium pH. Panels A and B in Fig. 5 demonstrate RLS spectra for C1 (Panel A) and C7 (Panel B) obtained at the different pH values of the medium. As reported in the literature (mainly by Pasternack and Parkash [48, 49]), the appearance of RLS bands should primarily be associated with chromophore aggregation of the systems present in the solution. Analysis of all the RLS spectra shown in Fig. 5 reveals the presence of RLS spectra (with higher or lower intensity) in the pH ranges where the dual fluorescence effect is observed for C1 and C7, as presented above in Fig. 1 (Panels B and D). Additionally, it can be noted that the increase in the pH value is accompanied by reduction of the RLS signal for both analysed analogues. Panel C in Fig. 5 shows the relationship between the RLS signal intensity and the solution pH for C1 (black line) and C7 (grey line). As can be noted, the RLS signal intensity substantially declines with the increase in pH, which clearly suggests an impact of molecular aggregation on the spectral effects. The intensity of the signals is clearly higher for C7, where a substantial decline in the absorbance level was revealed by the measurements of absorption spectra. This evidences the impact of the alkyl substituent structure on the strength of the aggregation interactions in C7. The RLS signal loses its intensity at a pH value of ca. 7 for C7 and C1. The presence of the RLS spectra and their dependence on the changes in the pH of the analysed solutions clearly proves a relationship between the presented effect and the molecular aggregation of the investigated 1,3,4-thiadiazoles. It is also worth mentioning that the oscillatory structure of the RLS bands evidences the presence of various possibly different-size aggregation structures of both C1 and C7, which also confirms the impact of the structure of the analysed analogues, in particular their alkyl substituents, on the observed effect (Scheme 1).

The observations clearly indicate a greater impact of molecular aggregation effects than the aforementioned TICT, ESIPT, and anti-Kasha processes or excimer fluorescence. Additionally, aggregation has a significant impact on changes in the electron charge distribution around the analysed molecules, which yields fluorescence spectral effects in a pH range. The difference in the structure of substituent groups of the analysed analogues (C1, C7) evokes different aggregation effects. Therefore, we postulate that the dual fluorescence effects in the 1,3,4-thiadiazoles are induced by at least two overlapping effects, i.e. molecular aggregation (depending on the substituent type in the molecule) and charge transfer CT (induced by the aggregation factor). Based on the spectral shifts in the electron absorption spectra, formation of *card pack* rather than *head to tail* aggregates can be assumed. Furthermore, the aggregation can be stronger at low, neutral, or very high pH values, whereas additional intermediate forms, which are a resultant of both ionised forms, can appear at neutral pH. This can explain the rapid decline in the intensity of the RLS signal at neutral and high pH values. These considerations will be continued in further investigations of this highly attractive dual fluorescence spectroscopic effect in the 1,3,4-thiadiazole group with a resorcyl ring in the structure.

## Conclusions

The results presented in this paper indicate appearance of the dual fluorescence effect in the fluorescence emission spectrum of the analysed 1,3,4-thiadiazole analogues C1 and C7 in the aqueous medium. In both compounds, this effect is most evident at the physiological pH and values lower that ca. 7. Furthermore, the comparison of the analysed analogues with the previously described FABT compound, differing structurally by the presence of the amino –N-H group in the substituent group and the fluorobenzene ring, revealed that there was no longwave fluorescence band with a maximum at ca. 500 nm. In the case considered in this paper, a shortwave fluorescence emission band with a maximum at ca. 380 nm was found to accompany the decline in the pH value. This effect may be related to the structural differences (absence of the N-H group in the C1 and C7 structure) between the compounds. In another study of a compound that is structurally similar to FABT but does not comprise the fluorobenzene fragment and differs from C1 only in the presence of the N-H amine group, a band with a maximum at ca. 380 nm was observed at high pH values (results submitted for publishing). Acidification of the medium yielded a separate band at ca. 440 nm (as in the case of C1 and C7 at the high pH values). Therefore, there is an evident impact of the substituents in the 1,3,4-thiadiazole structure on the position of fluorescence bands despite the similar/identical chromophore organisation (five conjugated double bonds) in the analysed molecules. The lengthened fluorescence lifetime and change in the intensity of the RLS spectrum in the specified pH range for both molecules indicate association of this effect with the aggregation phenomenon dependent on the alkyl chain length. Therefore, it has been proposed that probably a combination of two effects, i.e. molecular aggregation (with the form dependent on the analogue) and CT charge transfer, is responsible for the observed fluorescence phenomena. This hypothesis is largely confirmed by the quantum-mechanical calculations (TDDFT) performed for other analogues from this group of compounds. The non-specific interactions (e.g. formation of intra- and intermolecular hydrogen bonds or CT states) in the analysed 1,3,4-thiadiazole analogues modify the electronic structure surrounding the molecule, leading to changes in the distribution of electron density and thereby forcing CT charge transfer in the analysed analogues. This effect is reversible after alkalinisation of the medium and at high pH values (single fluorescence) and after acidification of the medium at pH in the range from 1 to ca. 7 (dual fluorescence).

The dual fluorescence effect occurs in a different energetic range than that in the 1,3,4-thiadiazoles analysed previously. In the theoretical aspect, it can be used for analysis of excitation states in these and other structurally similar molecules and for designing new fluorescence probes.

In conclusion, it should be emphasised that the analysed 1,3,4-thiadiazoles can serve as excellent fluorescent probes that are highly sensitive to changes in the medium pH. Additionally, the attractiveness of the investigated systems is enhanced by their advantages in medical and pharmacological applications, which are more often based on new compounds with desirable properties.

## Electronic supplementary material

ESM 1 (DOC 106 kb)
