# Supplementary material for: Spectroscopic Studies of Fluorescence Effects in Bioactive 4-(5-Heptyl-1,3,4-Thiadiazol-2-yl)Benzene-1,3-Diol and 4-(5-Methyl-1,3,4-Thiadiazol-2-yl)Benzene-1,3-Diol Molecules Induced by pH Changes in Aqueous Solutions
Source: J Fluoresc. 2017 Mar 1;27(4):1201–12. doi: 10.1007/s10895-017-2053-y (PMC5487764; doi:10.1007/s10895-017-2053-y)
Supplement: Supplementary file 1 — (DOC 106 kb) [file 10895_2017_2053_MOESM1_ESM.doc]

**Spectroscopic Studies of Fluorescence Effects in bioactive 4-(5-heptyl-1,3,4-thiadiazol-2-yl)benzene-1,3-diol and 4-(5-methyl-1,3,4-thiadiazol-2-yl)benzene-1,3-diol molecules Induced by pH Changes in Aqueous Solutions**

Arkadiusz Matwijczuk^1*^, Dariusz Kluczyk^5^, Andrzej Górecki^3^, Andrzej Niewiadomy^2,4^, and Mariusz Gagoś^5*^

1) Department of Biophysics, University of Life Sciences in Lublin, Akademicka 13, 20-950

Lublin, Poland

2) Department of Chemistry, University of Life Sciences in Lublin, 20-950 Lublin, Poland

3) Department of Physical Biochemistry, Faculty of Biochemistry, Biophysics and Biotechnology of the Jagiellonian University, Gronostajowa 7, 30-387 Krakow, Poland

4) Institute of Industrial Organic Chemistry, Annopol 6, 03-236 Warsaw, Poland

5) Department of Cell Biology, Institute of Biology, Maria Curie-Skłodowska University, 20-

033 Lublin, Poland

Corresponding authors:

Arkadiusz Matwijczuk

Department of Biophysics

University of Life Sciences in Lublin 20-950 Lublin,

Fax: +(48 81) 4456684, Phone: +(48 81) 445 69 37

E-mail: arkadiusz.matwijczuk@up.lublin.pl, arekmatwijczuk@gmail.com

Mariusz Gagoś

Department of Cell Biology

Institute of Biology and Biotechnology, Maria Curie–Skłodowska University

20–033 Lublin, Poland.

Phone: +(48 81) 537 59 04

E–mail: mariusz.gagos@poczta.umcs.lublin.pl


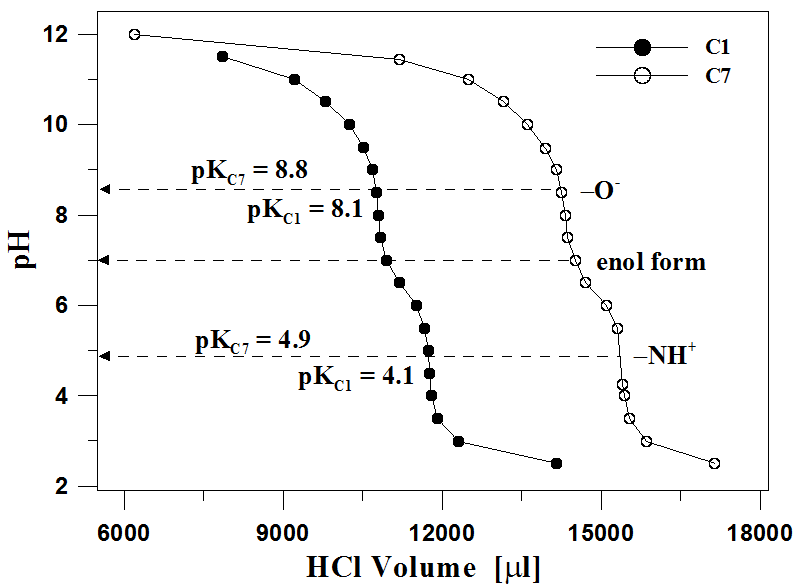


**Fig. S1.** pH-metric titration curves for C1 (black circles) and C7 (white circles). Titration was carried out with the use of 0.1 M HCl. The insets present groups undergoing ionisation in the resorcyl ring (–O^-^ group) and in the 1,3,4-thiadiazole ring (–NH^+^ group). Dotted lines denote points pK for both compounds and pH 7 (pH, at which neutral forms of both compounds dominate). For the –O^-^ group at the *ortho* position in the resorcyl ring pK_C7_=8.8 and pK_C1_=8.1 (figure insets) and for the –NH^+^ group pK_C7_=4.9 and pK_C1_=4.1 (figure insets).


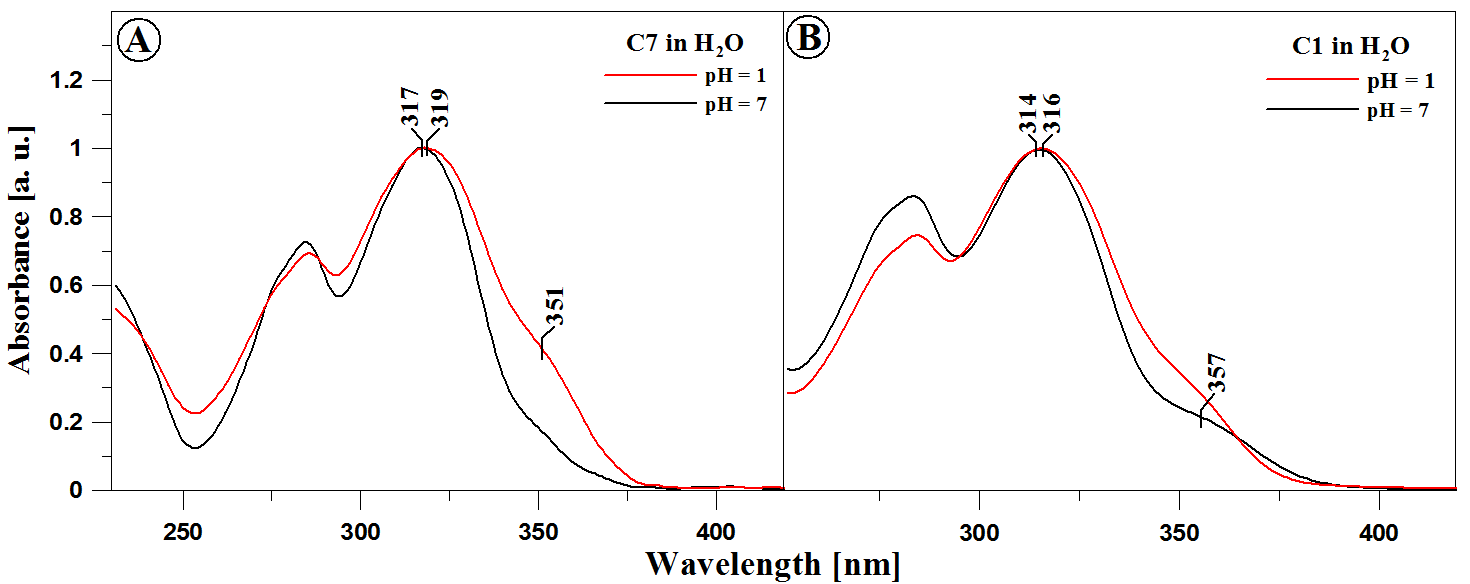


Fig. S2. Electron absorption spectra for C7 (Panel A) and C1 (Panel B) at pH 7 (black lines in both panels) and at pH 1 (red lines in both panels) normalised at the maximum. The widths of spectral slits are specified in the Material and Methods; all measurements were performed at 22 ^o^C.

**Table S1.** Values of the molar extinction coefficient and the transition dipole moment for C1 and C7 ^1^.

| **C1** |  |  | **C7** |  |
| --- | --- | --- | --- | --- |
| **Solvents** | ε [M^-1^*cm^-1^] | µ [Deby] | ε [M^-1^*cm^-1^] | µ [Deby] |
| DMSO | 14576.91 | 3.85 | 16638.97 | 4.13 |
| Methanol | 12531.75 | 3.77 | 14136.16 | 3.93 |
| Ethanol | 13756.65 | 3.89 | 14698.89 | 4.02 |
| 2-propanol | 14257.45 | 4.93 | 14982.26 | 4.12 |
| Butanol | 15173.54 | 4.14 | 15601.43 | 4.22 |
| ACN | 11082.22 | 3.40 | 12834.31 | 3.67 |
| DMF | 13776.74 | 3.81 | 14007.11 | 3.91 |
| Chloroform | 12332.79 | 3.63 | 13930.36 | 3.80 |
| H_2_O | - | 4.16 | - | 4.25 |

**References**

1. Matwijczuk, A.; Kluczyk, D.; Górecki, A.; Niewiadomy, A.; Gagos, M., Solvent Effects on Molecular Aggregation in 4-(5-heptyl-1,3,4-thiadiazol-2-yl)benzene-1,3-diol and 4-(5-methyl-1,3,4-thiadiazol-2-yl)benzene-1,3-diol*. The Journal of Physical Chemistry* **B 20**16.
